# Human papillomavirus infections during pregnancy and adverse pregnancy outcomes: a Scandinavian prospective mother-child cohort study

**DOI:** 10.1186/s12884-024-06958-2

**Published:** 2024-11-19

**Authors:** Magdalena R. Værnesbranden, Anne Cathrine Staff, Johanna Wiik, Katrine Sjøborg, Corina S. Rueegg, Meryam Sugulle, Karin C. Lødrup Carlsen, Berit Granum, Guttorm Haugen, Gunilla Hedlin, Katarina Hilde, Björn Nordlund, Eva M. Rehbinder, Knut Rudi, Håvard O. Skjerven, Birgitte K. Sundet, Cilla Söderhäll, Riyas Vettukattil, Christine M. Jonassen

**Affiliations:** 1 Department of Obstetrics and Gynecology, Østfold Hospital Trust, P.O. Box 300, Kalnes, Kalnes 1714 Norway; 2 Faculty of Medicine, Institute of Clinical Medicine, University of Oslo, P.O. Box 1702, Blindern, Oslo, 0316 Norway; 3 Division of Obstetrics and Gynaecology, Oslo University Hospital, Oslo, Norway; 4 Department of Obstetrics and Gynecology, Institute of Clinical Sciences, Sahlgrenska Academy, Gothenburg University, Gothenburg, Sweden; 5 Oslo Centre for Biostatistics and Epidemiology, Oslo University Hospital, Oslo, Norway; 6 Division of Paediatric and Adolescent Medicine, Oslo University Hospital, Oslo, Norway; 7 Department of Chemical Toxicology, Norwegian Institute of Public Health, Oslo, Norway; 8 Department of Dermatology and Vaenereology, Oslo University Hospital, Oslo, Norway; 9 Astrid Lindgren Children’s Hospital, Karolinska University Hospital, Stockholm, Sweden; 10 Department of Women’s and Children’s Health, Karolinska Institutet, Stockholm, Sweden; 11 Faculty of Chemistry, Biotechnology and Food Science, Norwegian University of Life Sciences, Ås, Norway; 12 Genetic Unit, Centre for Laboratory Medicine, Østfold Hospital Trust, Kalnes, Norway; 13 Department of Virology, Norwegian Institute of Public Health, Oslo, Norway

**Keywords:** Human papillomavirus and pregnancy, Placental dysfunction syndromes, Hypertensive disorders of pregnancy, Gestational diabetes mellitus, Small for gestational age

## Abstract

**Background:**

Human papillomaviruses are common in the urogenital tract amongst women of childbearing age. A few studies indicate a possible association between human papillomavirus infections in pregnancy and adverse pregnancy outcomes whilst other studies find no such association. We aimed to investigate the association between human papillomavirus infections during pregnancy and adverse pregnancy outcomes linked to placental dysfunction, including hypertensive disorders of pregnancy, gestational diabetes mellitus and newborns small for gestational age.

**Materials and methods:**

Pregnant women from the general population in Norway and Sweden were enrolled at the time of routine mid-gestational ultrasound examination. Urine samples collected at mid-gestation in 950 and at delivery in 753 participants, were analyzed for 28 human papillomavirus genotypes, including 12 high-risk genotypes. Participants completed electronic questionnaires at enrollment and medical records were reviewed for background characteristics and for the following adverse pregnancy outcomes: hypertensive disorders of pregnancy including gestational hypertension, preeclampsia, superimposed preeclampsia, eclampsia and Hemolysis Elevated Liver enzymes and Low Platelets (HELLP) syndrome, gestational diabetes mellitus, and newborns small for gestational age. Associations between adverse pregnancy outcomes and (a) any human papillomavirus, high-risk human papillomavirus and human papillomavirus genotype 16 infection at mid-gestation, (b) multiple genotype infections at mid-gestation, and (c) persisting infections during pregnancy were assessed with univariable and multivariable logistic regression models. Missing covariates were imputed using multiple imputation.

**Results:**

At mid-gestation, 40% (377/950) of women were positive for any of the 28 genotypes, 24% (231/950) for high-risk genotypes and human papillomavirus 16 was found in 6% (59/950) of the women. Hypertensive disorders of pregnancy was observed in 9% (83/950), gestational diabetes mellitus in 4% (40/950) and newborns small for gestational age in 7% (67/950). Human papillomavirus infection with any genotype, high-risk or human papillomavirus genotype 16 at mid-gestation was not associated with adverse pregnancy outcomes. No associations were found for multiple genotype infections at mid-gestation or persisting infections.

**Conclusion:**

In a general population of pregnant women, we found no evidence of human papillomavirus infections during pregnancy being associated with hypertensive disorders of pregnancy, gestational diabetes mellitus, or newborns small for gestational age.

**Trial registration:**

Trial registration The study is registered at ClincialTrials.gov; NCT02449850 on May 19th, 2015.

**Graphical Abstract:**

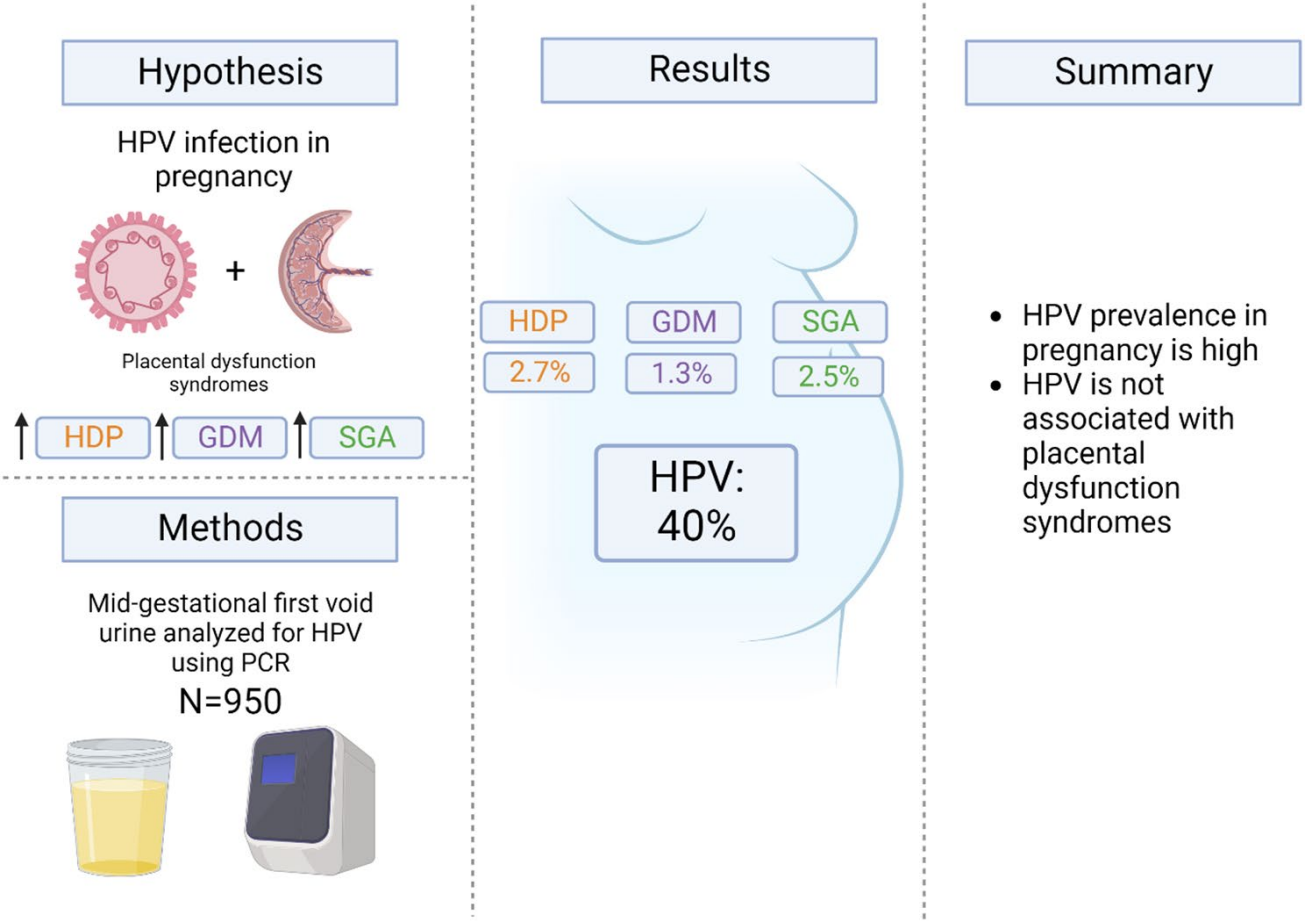

**Supplementary Information:**

The online version contains supplementary material available at 10.1186/s12884-024-06958-2.

## Introduction

The role of human papillomaviruses (HPVs) for human health has been extensively investigated. Its etiological role in cervical dysplasia is well established [1], while associations with adverse pregnancy outcomes are less clear [2–4]. The prevalence of HPV infections peaks during the childbearing years of women, hence being the most common sexually transmitted infection. Several studies have demonstrated the presence of HPV in the placenta, specifically in trophoblast cells [5–7]. Further, human papillomavirus has been shown to replicate in cultivated trophoblasts [5–9]. In addition to this, HPV reduces adhesion and migratory properties as well as the number of trophoblast cells [8, 10]. As extracellular trophoblast invasion is crucial for placentation and subsequent placental function, it is important to assess whether obstetric syndromes that are mediated by placental dysfunction are more prevalent in pregnancies affected by HPV infection. As HPV has been detected in first trimester placentae [5, 11, 12], HPV infection could potentially lead to placental dysfunction and thereby adverse pregnancy outcomes. Hypertensive disorders of pregnancy (HDP) include gestational hypertension (GH), preeclampsia, superimposed preeclampsia, eclampsia and Hemolysis Elevated Liver enzymes and Low Platelets (HELLP) syndrome. These disorders are closely linked to placental dysfunction and represent major causes of maternal and perinatal mortality and morbidity as well as increased risk of long-term cardiovascular disease in both mother and offspring [13–19]. Gestational diabetes mellitus (GDM), also linked to placental dysfunction, increases the risk for additional adverse pregnancy outcomes, and neonatal adverse outcomes [20, 21]. Women with GDM have an increased risk for developing postpartum diabetes mellitus type II, and long-term cardiovascular disease [17, 22]. Small for gestational age (SGA) represents a proxy for fetal growth restriction, also representing a clinical feature of placental dysfunction [23]. Newborns born SGA are at risk for both short- and long-term health complications [17].

Research on adverse pregnancy outcomes and HPV infections has mainly focused on miscarriage or preterm delivery and to a lesser degree on adverse outcomes linked to placental dysfunction. An association between HPV and HDP, or SGA has been reported by some studies [4, 24–26], however, others find no association [27–29]. Overall, studies are inconclusive and incomparable, due to differing methodologies, non-uniform definitions of adverse outcomes, and small sample sizes. Some studies are retrospective or data-linkage studies [25, 26, 28, 29], and some use abnormal Papanicolaou (PAP) smears as a proxy for HPV infections [25, 26, 29]. A review and meta-analysis by Niyibizi et al., concluded that many studies are of poor quality and further investigations are necessary [24].

Using internationally recognized definitions for HDP, GDM and SGA in a prospective cohort of pregnant women from the mother-child PreventADALL (Preventing Atopic Dermatitis and Allergies in children) study [30], we aimed to investigate whether HPV infections during pregnancy were associated with adverse pregnancy outcomes linked to placental dysfunction.

## Materials and methods

The present study is a substudy of the multi-center prospective mother-child cohort study. The PreventADALL study aims to investigate early life interventions as a possible prevention for allergic diseases as well as to identify early life factors, including factors during pregnancy and delivery, associated with non-communicable diseases (NCD) [30]. Briefly, pregnant women from the general population were invited to participate in the PreventADALL study at the time of their second trimester routine ultrasound examination, excluding women who were not proficient in Norwegian or Swedish, were pregnant with more than two fetuses or had a severely diseased fetus. Women were asked to complete comprehensive electronic (e-) questionnaires at gestational weeks (GW) 18 and 34. Further study details are described elsewhere [3, 30]. Participants in the PreventADALL study were recruited from December 2014 to October 2016 and enrolled at Oslo University Hospital and Østfold Hospital Trust (Norway), as well as Karolinska Institute, Stockholm (Sweden) [30]. Pregnant women who provided first-void urine samples at the time of inclusion (second trimester routine ultrasound scan) and at delivery were included in the present substudy.

**Fig. 1:**
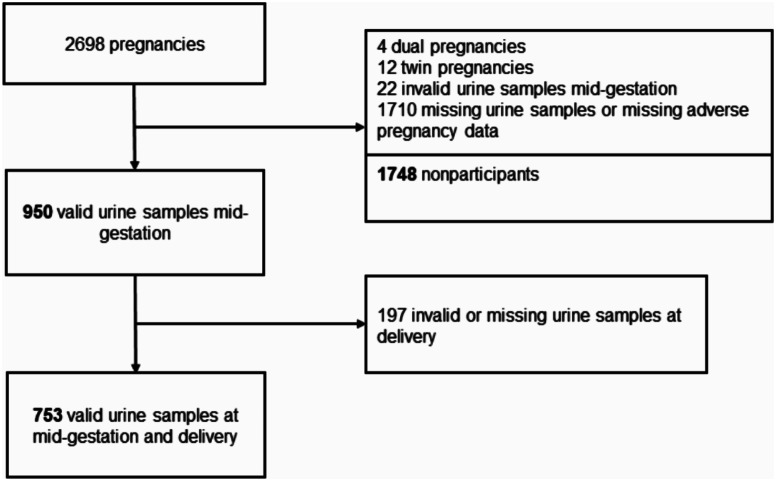
Flow chart of enrolled women in the current study

Of the 2701 pregnancies included, twin pregnancies (*n* = 12) were excluded, and in the case of women participating with two separate pregnancies (*n* = 4), the pregnancy with a urine sample with a valid HPV result at mid-gestation and delivery was included in the analyses. If both pregnancies had valid urine samples, the first pregnancy was selected. Women missing all adverse pregnancy outcome data were excluded, yielding 950 women that provided urine samples with a valid HPV result at the time of enrollment (GW16-22, mid-gestation), of whom 753 women had urine samples with valid HPV result at both mid-gestation and delivery (Fig. 1 and Supplementary Fig. 1). Pregnancy outcomes were recorded through medical chart revision in Norway and from birth registries in Sweden [31, 32]. Women were asked to provide first-void urine samples at mid-gestation and delivery. Urine was collected and kept frozen at -80 °C until analysis. Detection and genotyping of HPV DNA was performed using the Seegene Anyplex II HPV28 detection polymerase chain reaction (PCR) assay (Seegene Inc, Seoul, South Korea). Detailed description of urine handling and analysis has previously been described [3]. Seegene Anyplex II HPV28 kit detects and genotypes 28 genital HPV genotypes, defined as Any-HPV group (HPV16, 18, 31, 33, 35, 39, 45, 51, 52, 56, 58, 59, 26, 53, 66, 68, 69, 70, 73, 82, 6, 11, 40, 42, 43, 44, 54, 61). Human papillomavirus genotypes were further subclassified according to their malignant potential, as high-risk (HR-) HPV (HR-HPV16, 18, 31, 33, 35, 39, 45, 51, 52, 56, 58, 59), according to the International Agency of Research on Cancer. Analyses were also performed with HPV16, which has the highest malignant potential [33, 34].

Exposures in this study were recorded at mid-gestation and, in the case of persistence, at mid-gestation and delivery. The exposure variables were the detection of Any-HPV, HR-HPV, HPV16 infection or multiple infections at mid-gestation, as well as genotype specific persistence of Any-HPV, HR-HPV or HPV16 from mid-gestation to delivery. We defined three groups of multiple HPV infections:

1. Multiple infections with Any-HPV defined as having ≥ 2 different HPV genotypes.
2. Multiple infections with HR-HPV defined as having ≥ 2 different HPVs, with at least one HR-HPV genotype.
3. Multiple HPV16 infections defined as having HPV16 with any other genotype.

Persistence of Any-HPV, HR-HPV or HPV16 infection during pregnancy was defined as having the same genotype-specific HPV infection at mid-gestation and delivery. This definition was based on the long clearance time of HPV and the low probability of a woman engaging with a new sexual partner during her late pregnancy, thus contracting a new HPV infection of the same genotype as at mid-gestation.

The outcomes of this study are ongoing processes that occur during the pregnancy but were recorded at the time of delivery. The adverse pregnancy outcomes were defined as:

1. Hypertensive disorders of pregnancy including:
  - Gestational hypertension: new-onset hypertension (systolic blood pressure ≥ 140mmHg and/or diastolic blood pressure ≥ 90mmHg) occurring from or after GW 20, without proteinuria.
  - Preeclampsia: gestational hypertension with new-onset proteinuria.
  - Superimposed preeclampsia: chronic hypertension with new onset of proteinuria after GW 20.
  - Eclampsia: General tonic clonic seizures occurring in women with preeclampsia.
  - HELLP syndrome: defined according to clinical guidelines and based on blood analyses [32].

Hypertensive disorders of pregnancy subgroups were defined according to the commonly used definitions at the time of study recruitment (2014-16) [32], and did in our study not include women with preexisting hypertension or hypertension diagnosed prior to GW 20 (chronic hypertension).

2) Gestational diabetes mellitus, defined as glucose intolerance diagnosed during pregnancy [22], was laboratory-wise defined according to clinical guidelines relevant at the time of study inclusion (2014-16). An oral glucose intolerance test with 75 g oral glucose was administered and an overnight fasting glucose level < 7.0mmol/L and 2 h glucose level ≥ 7.8 (but < 11.1mmol/L) confirmed a GDM diagnosis [35].
3) Newborns small for gestational age were defined as birthweight below the 10th percentile for gestational age, stratified according to sex [36].

Possible confounding factors were collected from e-questionnaires including maternal age at enrollment, pre-pregnancy body mass index (ppBMI), duration of pre-conception relation with the child’s father (< 1 year, 1–2 years, 3–4 years or > 5 years), parity (0, 1 or ≥ 2), nicotine use during pregnancy (yes or no), alcohol consumption during pregnancy (yes or no), maternal chronic disease including chronic hypertension and pregestational diabetes mellitus (yes or no) and maternal educational level (preliminary/high school only, higher education ≤ 4 years or higher education/PhD > 4 years).

### Statistical analyses

**Fig. 2:**
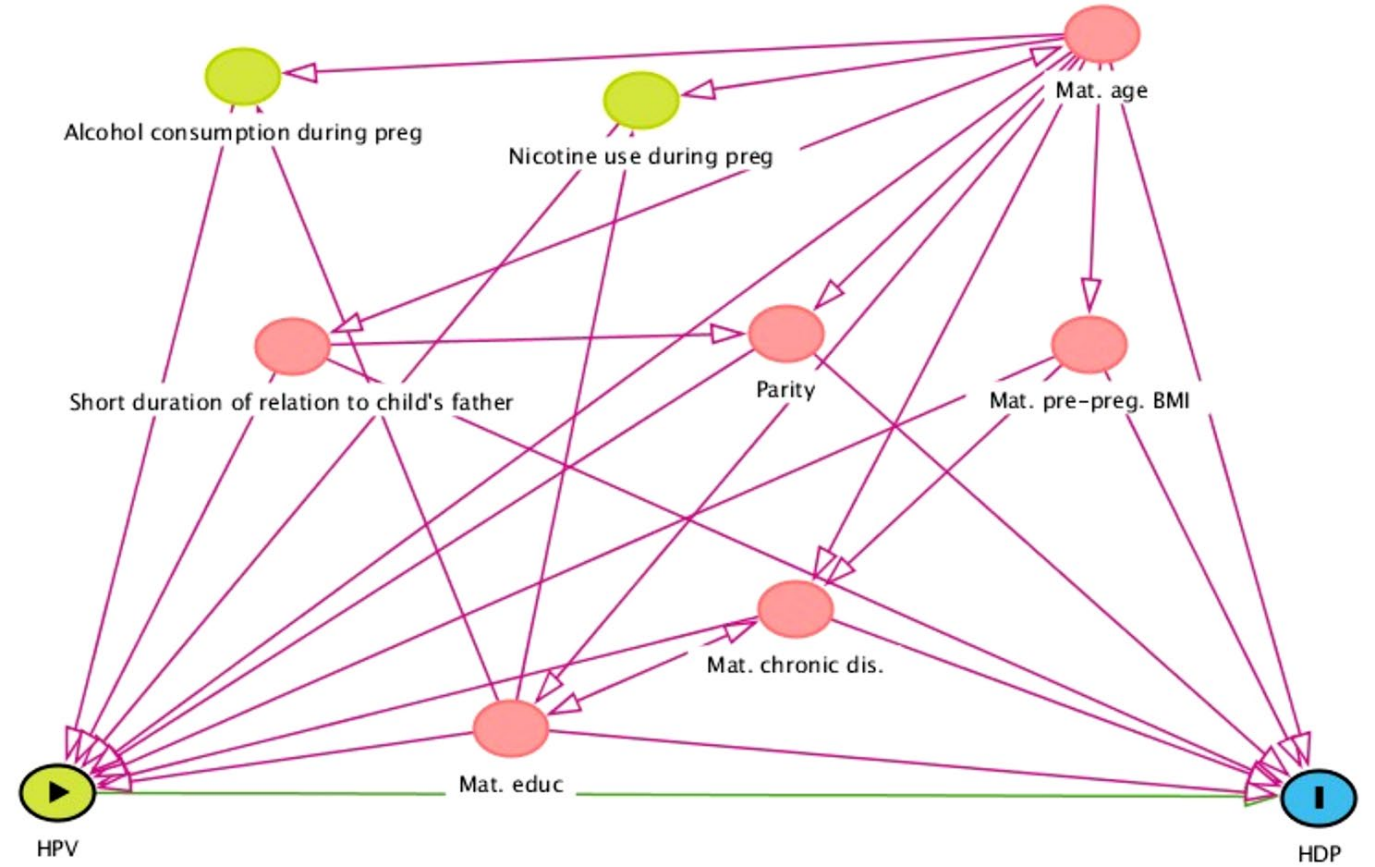
Directed acyclic graph of covariates of hypertensive disorders of pregnancy. Abbreviations HDP- hypertensive disorders of pregnancy, HPV-human papillomavirus, Mat.edu-maternal education, Mat.chornic dis.-maternal chronic disease, including chronic hypertension and pregestational diabetes mellitus, Mat.pre-preg.BMI- maternal pre-pregnancy Body Mass Index, Mat.age-maternal age, preg-pregnancy. Color explanation: green with arrow-exposure, blue with l- outcome of interest, green- ancestor (covariate) of exposure, blue- ancestor of outcome, pink-covariates needed for the adjustment analyses

**Fig. 3:**
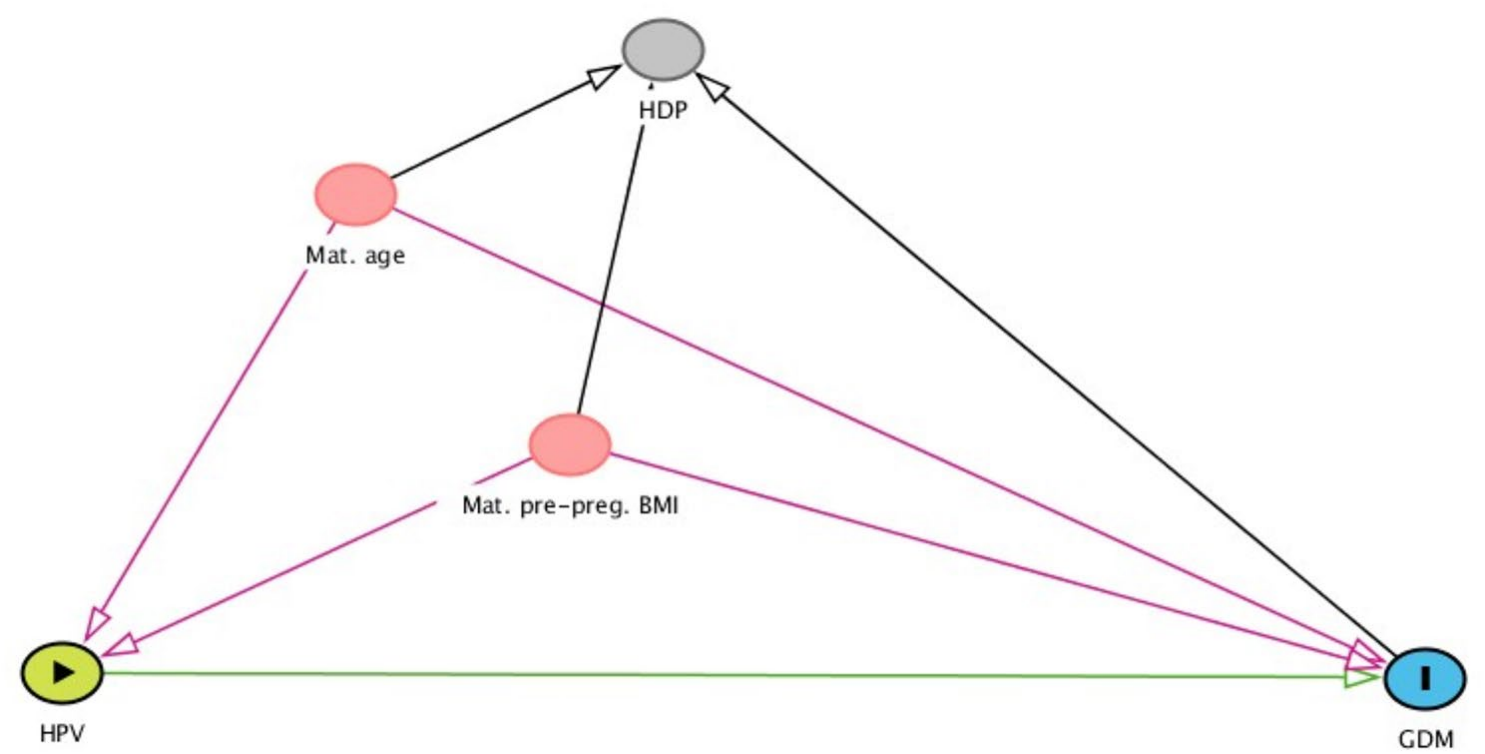
Directed acyclic graph of covariates for gestational diabetes mellitus. Abbreviations GDM-gestational diabetes mellitus, HPV-human papillomavirus, Mat.pre-preg.BMI- maternal pre-pregnancy Body Mass Index, Mat.age-maternal age. Color explanation: green with arrow-exposure, blue with l- outcome of interest, pink-covariates needed for the adjustment analyses, grey- other variable

**Fig. 4:**
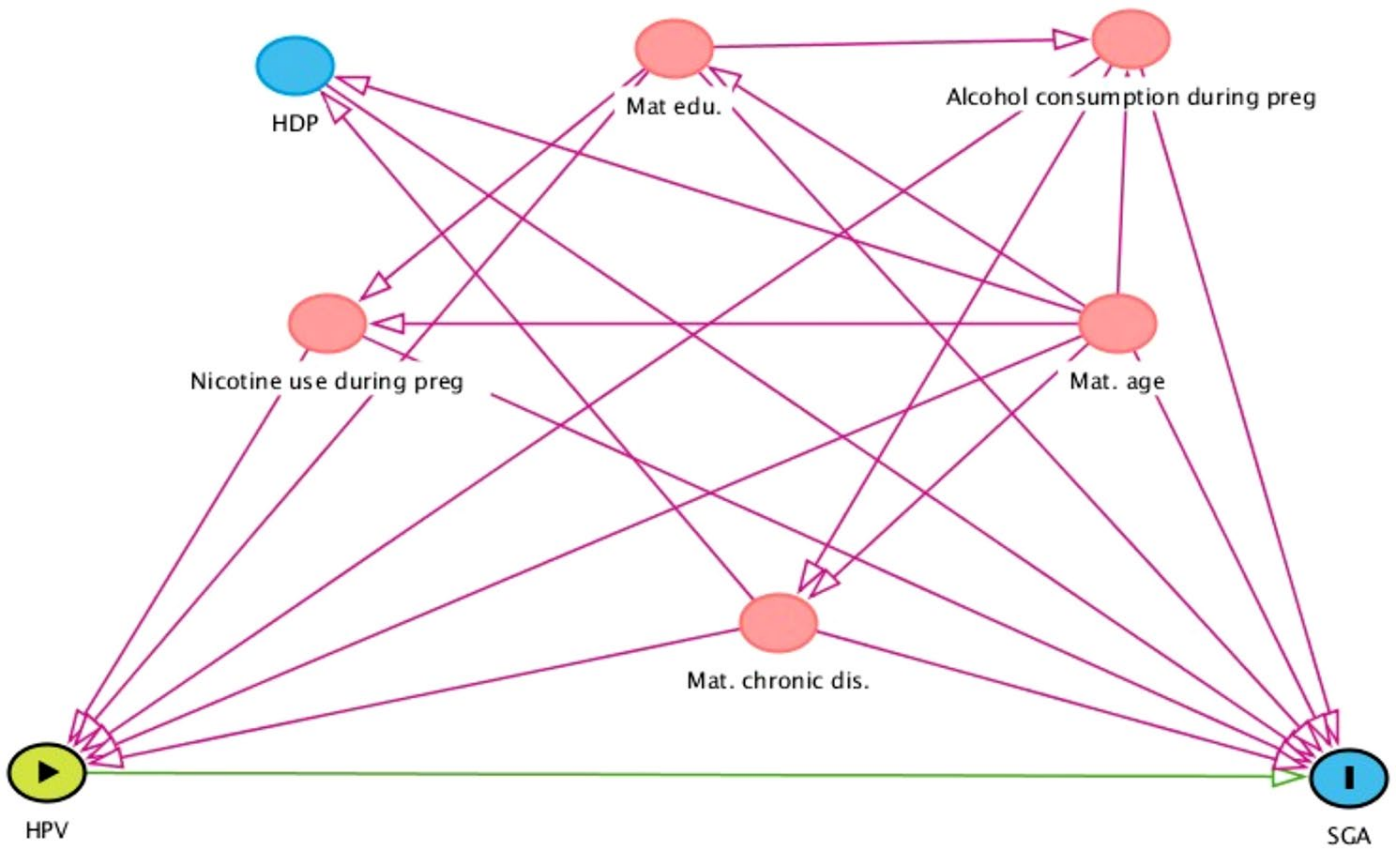
Directed acyclic graph of covariates of newborn small of gestational age. Abbreviations: SGA- newborn of small gestational age, HPV-human papillomavirus, Mat.edu-maternal education, Mat.chornic dis.-maternal chronic disease, including chronic hypertension and pregestational diabetes mellitus, Mat.age-maternal age, preg-pregnancy, HDP- hypertensive disorders of pregnancy. Color explanation: green with arrow-exposure, blue with l- outcome of interest, blue- ancestor (covariate) of outcome, pink-covariates needed for the adjustment analyses

Categorical variables are presented with numbers and percentages and continuous variables as means with standard deviations (SD) or medians with minimum and maximum values. Univariable and multivariable logistic regression models were used to investigate the association between Any-HPV, HR-HPV, HPV16 or multiple HPV infection at mid-gestation as well as persistent infection of Any-HPV, HR-HPV or HPV16 and adverse pregnancy outcomes. We ran separate models for each exposure variable and each of the three outcomes. Multivariable regression models were adjusted for potential confounders based on three directed acyclic graphs [37] (DAGs; Figs. 2, 3 and 4). In cases where cells contained < 5, univariable exact logistic regression models were done. The multivariable exact logistic regression models did not converge therefore multivariable logistic regression models were performed. Missing covariate values added up to 13–16% and were imputed using multiple imputation with chained equations and 40 data sets were imputed. Results presented regarding associations are from the adjusted imputed model.

Statistical analyses were performed using IBM© SPSS© statistics version 28 (Chicago, IL, U.S.A) or Stata version 16 (StataCorp LLC, College Station, Texas). A P-value < 0.05 was considered statistically significant.

## Results

At the time of enrollment, the mean age of the baseline sample (*N* = 950) was 32.0 (SD = 4.6) years and median ppBMI 24.5 kg/m^2^ (min-max: 17.8–48.2). Most women were married, 87% (825/950) with 52% (498/950) of the women in a relationship lasting 5 years or longer. Most women were expecting their first child, 55% (518/950). Mean gestational age at delivery was 40.1 weeks (SD = 1.5) and mean birthweight 3592 g (SD = 491.1; Table 1). No major differences were observed between the baseline sample (*N* = 950) and nonparticipants (*N* = 1748; Table 1). In the baseline sample, 40% (377/950) were infected with Any-HPV whereas 24% (231/950) had an infection with HR-HPV. The most common single HPV genotype was HPV16 with 6% (59/950). Persistence of Any-HPV was 52% (152/290) and 52% (93/178) for HR-HPV whilst 64% (29/45) of HPV16 infections persisted until delivery.

**Table 1:** Maternal characteristics for women in baseline sample (*N* = 950) and nonparticipants (*N* = 1748)

	Total *n*. 950	HR-HPV^a^ positive *n*. 231	HR-HPV^a^ negative *n*. 719	Any-HPV^b^ positive *n*. 377	Any-HPV^b^ negative *n*. 573	Nonparticipants from PreventADALL study *n*. 1748
Maternal Age, yr. Mean (SD)	32.0 (4.6)	31.3 (4.6)	32.3 (4.3)	31.7 (4.6)	32.2 (4.2)	32.5 (4.0)
Maternal ppBMI. Median (min-max)	24.5 (17.8–48.2)	24.7 (18.7–38.9)	24.5 (17.8–48.2)	24.7 (18.4–39.3)	24.4 (17.8–48.2)	23.9 (17.2–44.2)
Marital Status. n (%)						
Married/cohabitants	825 (87%)	199 (96%)	626 (99%)	322 (96%)	503 (99%)	1465 (84%)
Single/Divorced/Other	17 (18%)	9 (4%)	8 (1%)	13 (4%)	4 (1%)	50 (3%)
Missing	108					233
Maternal education. n (%)						
Preliminary/high school only	141 (15%)	47 (23%)	94 (15%)	72 (22%)	69 (14%)	118 (7%)
Higher education ≤ 4 years	295 (31%)	69 (34%)	226 (36%)	112 (24%)	183 (36%)	462 (26%)
Higher/PhD/> 4 years	400 (42%)	89 (43%)	311 (49%)	148 (45%)	252 (50%)	923 (53%)
Missing	114					245
Duration of pre-conception relation with child’s father, yr. n (%)						
<1	27 (3%)	11 (6%)	16 (3%)	14 (5%)	13 (3%)	69 (4%)
1–2	89 (9%)	43 (23%)	46 (8%)	64 (21%)	25 (5%)	199 (11%)
3–5	188 (20%)	64 (34%)	124 (20%)	92 (30%)	96 (20%)	382 (22%)
>5	498 (52%)	71 (38%)	427 (70%)	141 (45%)	357 (73%)	806 (46%)
Missing	148					292
Nicotine use during pregnancy. n (%)						
No	740 (78%)	176 (85%)	564 (89%)	289 (86%)	451 (89%)	1327 (76%)
Yes	100 (11%)	31 (15%)	69 (11%)	45 (14%)	55 (11%)	179 (10%)
Missing	110					242
Alcohol consumption during pregnancy. n (%)						
No	591 (62%)	134 (65%)	457 (73%)	224 (67%)	367 (73%)	919 (53%)
Yes	245 (26%)	72 (35%)	173 (27%)	109 (33%)	136 (27%)	582 (33%)
Missing	114					247
Number of previous deliveries (Parity). n (%)						
0	518 (55%)	161 (70%)	357 (50%)	234 (62%)	284 (50%)	1075 (62%)
1	321 (34%)	55 (24%)	266 (37%)	111 (30%)	210 (37%)	539 (31%)
≥2	109 (11%)	15 (6%)	94 (13%)	31 (8%)	78 (14%)	128 (7%)
Missing	2					6
Gestational age at birth, weeks. Mean (SD)	40.1 (1.5)	40.1 (1.4)	40.1 (1.5)	40.1 (1.3)	40.0 (1.5)	39.9 (1.8)
Fetal sex. n (%)						
Male	495 (52%)	115 (50%)	380 (53%)	185 (49%)	310 (54%)	916 (52%)
Female	454 (48%)	116 (50%)	338 (47%)	191 (51%)	263 (46%)	811 (46%)
Missing	1					21
Birthweight, g. Mean (SD)	3591.8 (491.1)	3588.2 (468.1)	3574.0 (507.5)	3576.8 (453.0)	3577.8 (525.5)	3517.5 (559.0)
Blood pressure, mmHg. Mean (SD)						
Early pregnancy sBP (at inclusion)	110.3 (10.7)	110.6 (10.6)	110.4 (11.2)	110.3 (10.8)	110.5 (11.2)	107.9 (9.6)
Early pregnancy dBP (at inclusion)	63.1 (7.8)	63.4 (8.4)	63.6 (8.2)	62.8 (8.2)	64.0 (8.2)	61.0 (6.4)
Late pregnancy sBP (up to two weeks prior to delivery)	119.5 (12. 8)	120.5 (12.1)	119.1 (13.0)	119.0 (12.5)	119.7 (13.0)	120.4 (13.7)
Late pregnancy dBP (up to two weeks prior to delivery)	75.7 (9.8)	76.8 (9.7)	75.3 (9.8)	75.4 (9.8)	75.8 (9.8)	76.1 (9.6)
Maternal chronic disease (including chronic hypertension and pregest. diabetes mellitus). n (%)						
No	652 (69%)	153 (66%)	499 (69%)	258 (68%)	394 (69%)	1197 (69%)
Yes	298 (31%)	78 (34%)	220 (31%)	119 (32%)	179 (31%)	551 (31%)

^a^HR-HPV: 16, 18, 31, 33, 35, 39, 45, 51, 52, 56, 58, 59

^b^Any-HPV: 16, 18, 31, 33, 35, 39, 45, 51, 52, 56, 58, 59, 26, 53, 66, 68, 69, 70, 73, 82, 6, 11, 40, 42, 43, 44, 54, 61

Abbreviations: yr- years, SD- standard deviation, ppBMI- pre-pregnancy Body Mass Index, min-minimum, max-maximum, sBP-systolic blood pressure, dBP-diastolic blood pressure, g-grams. Pregest-pregestational

At the time of delivery, 9% (83/950) of the women were diagnosed with HDP (chronic hypertension not included). Gestational hypertension, diagnosed in 4% (42/950), was the most common HDP, followed by preeclampsia, 2% (19/950). None of the women developed eclampsia. Hypertensive disorders of pregnancy were observed in 7% (26/377) of women infected with Any-HPV and 8% (19/231) in women with HR-HPV at mid-gestation. In adjusted imputed logistic regression analyses, HDP was observed less often amongst women with Any-HPV infection at mid-gestation (adjusted odds ratio (aOR) 0.55, 95% confidence interval (CI) 0.32–0.93, *p* = 0.024), compared to women without infections; HR-HPV infections were not associated with HDP (aOR = 0.79, 95% CI 0.45–1.41, *p* = 0.427); Table 2). No associations were found in adjusted imputed logistic regression models for HPV16, multiple HPV infections at mid-gestation or persistent infections and HDP (Tables 2, 3 and 4, Supplementary Table 1). Four percent (40/950) of the women were diagnosed with GDM. The prevalence of GDM in women with Any-HPV was 3% (12/377) and 3% (8/231) in women with HR-HPV. We found no statistically significant association between Any-HPV infections or HR-HPV infections at mid-gestation and GDM in the adjusted imputed logistic regression models (aOR = 0.56, 95% CI 0.27–1.15, *p* = 0.114 and aOR = 0.77, 95% CI 0.34–1.75, *p* = 0.528, respectively; Table 2). No statistically significant associations were found for HPV16, multiple HPV infections at mid-gestation and persistent infections and GDM (Tables 2, 3 and 4, Supplementary Table 1). Overall, 7% (67/950) of the newborns were SGA, while SGA was observed in 6% (24/377) in women with Any-HPV infection and in 5% (11/231) of women with HR-HPV infection. No statistically significant associations were found between Any-HPV infection or HR-HPV infection at mid-gestation and SGA in the adjusted imputed logistic regression analysis (aOR = 0.76, 95% CI 0.45–1.29, *p* = 0.304 and aOR = 0.52, 95% CI 0.26–1.02, *p* = 0.058, respectively; Table 2). No statistically significant associations were found for HPV16, multiple HPV infections at mid-gestation and persistent infections and SGA (Tables 2, 3 and 4, Supplementary Table 1).

**Table 2:** HPV infection at mid-gestation and adverse pregnancy outcomes

HYPERTENSIVE DISORDERS OF PREGNANCY								
	Results from complete case analysis (*N* = 731)						Results from imputed analysis (*N* = 942)	
*N* = 942^a^	HPV positive	HDP^b^ case (HPV pos/neg) *N* = 83	Crude OR (95% CI)	p-value	aOR^c^ (95% CI)	p-value	Imputed aOR^d^ (95% CI)	p-value
Any-HPV^e^	375	26/57	0.67 (0.41–1.08)	0.100	0.67 (0.35–1.31)	0.242	0.55 (0.32–0.93)	**0.024**
HR-HPV^f^	231	19/64	0.91 (0.53–1.55)	0.718	0.83 (0.40–1.72)	0.621	0.79 (0.45–1.41)	0.427
HPV16	59	5/78	0.97 (0.29–2.50)	1.000	0.81 (0.23–2.78)	0.732	0.85 (0.32–2.26)	0.747
GESTATIONAL DIABETES MELLITUS								
	Results from complete case analysis (*N* = 883)						Results from imputed analysis (*N* = 903)	
*N* = 903^a^	HPV positive	GDM^g^ case (HPV pos/neg) *N* = 40	Crude OR (95% CI)	p-value	aOR^h^ (95% CI)	p-value	Imputed aOR^i^ (95% CI)	p-value
Any-HPV^e^	355	12/28	0.65 (0.33–1.30)	0.221	0.57 (0.28–1.17)	0.127	0.56 (0.27–1.15)	0.114
HR-HPV^f^	218	8/32	0.78 (0.35–1.71)	0.532	0.76 (0.34–1.73)	0.517	0.77 (0.34–1.75)	0.528
HPV16	57	2/38	0.79 (0.09–3.19)	1.000	0.83 (0.19–3.65)	0.808	0.85 (0.20–3.74)	0.834
NEWBORNS SMALL FOR GESTATIONAL AGE								
	Results from complete case analysis (*N* = 831)						Results from imputed analysis (*N* = 949)	
*N* = 949^a^	HPV positive	SGA^j^ case (HPV pos/neg) *N* = 67	Crude OR (95% CI)	p-value	aOR^k^ (95% CI)	p-value	Imputed aOR^l^ (95% CI)	p-value
Any-HPV^e^	376	24/43	0.84 (0.50–1.41)	0.510	0.79 (0.44–1.42)	0.437	0.76 (0.45–1.29)	0.304
HR-HPV^f^	231	11/56	0.59 (0.30–1.15)	0.121	0.60 (0.29–1.24)	0.167	0.52 (0.26–1.02)	0.058
HPV16	59	2/65	0.45 (0.05–1.76)	0.392	0.45 (0.11–1.94)	0.287	0.36 (0.09–1.55)	0.171

^a^Eight women with missing HDP data, 47 women with missing GDM data and 1 woman with missing SGA data

^b^Hypertensive disorders of pregnancy, including gestational hypertension, superimposed preeclampsia, preeclampsia, eclampsia, Hemolysis, Elevated Liver enzymes, Low Platelets (HELLP)

^c^Adjusted for: Duration of pre-pregnancy relation to child’s father, maternal age, pre-pregnancy Body Mass Index (ppBMI), parity, maternal chronic disease (including chronic hypertension and diabetes mellitus), maternal education

^d^Imputed variables: Maternal education, Duration of pre-pregnancy relation to child’s father, ppBMI

^e^HR-HPV: 16, 18, 31, 33, 35, 39, 45, 51, 52, 56, 58, 59

^f^Any-HPV: 16, 18, 31, 33, 35, 39, 45, 51, 52, 56, 58, 59, 26, 53, 66, 68, 69, 70, 73, 82, 6, 11, 40, 42, 43, 44, 54, 61

^g^GDM: Gestational Diabetes Mellitus

^h^Adjusted for: Maternal Age, ppBMI

^i^Imputed variables: ppBMI

^j^SGA-Newborns small for gestational age

^k^Adjusted for: Maternal chronic disease (including chronic hypertension and diabetes mellitus), nicotine use during pregnancy, alcohol consumption during pregnancy, maternal age. maternal education

^l^Imputed variables: Maternal education, nicotine use during pregnancy, alcohol consumption during pregnancy

Abbreviations: HPV-Human papillomavirus, HR-HPV-High-Risk Human papillomavirus, OR-Odds Ratio, aOR-adjusted Odds Ratio, pos-positive, neg-negative

**Table 3:** Multiple HPV infection at mid-gestation and adverse pregnancy outcomes

HYPERTENSIVE DISORDERS OF PREGNANCY								
	Results from complete case analysis (*N* = 731)						Results from imputed analysis (*N* = 942)	
*N* = 942^a^	HPV positive	HDP^b^ case (HPV pos/neg) *N* = 83	Crude OR (95% CI)	p-value	aOR^c^ (95% CI)	p-value	Imputed aOR^d^ (95% CI)	p-value
Any-HPV^e^ + Any-HPV	171	15/68	0.99 (0.55–1.79)	0.984	0.73 (0.32–1.68)	0.454	0.78 (0.41–1.50)	0.456
HR-HPV^f^ + Any-HPV	139	14/69	1.19 (0.65–2.18)	0.570	0.84 (0.36-2.00)	0.696	0.96 (0.49–1.84)	0.892
HPV16 + Any-HPV	38	4/79	1.24 (0.31–3.61)	0.857	1.38 (0.380–4.98)	0.627	1.04 (0.34–3.16)	0.942
GESTATIONAL DIABETES MELLITUS								
	Results from complete case analysis (*N* = 883)						Results from imputed analysis (*N* = 903)	
*N* = 903^a^	HPV positive	GDM^g^ case (HPV pos/neg) *N* = 40	Crude OR (95% CI)	p-value	aOR^h^ (95% CI)	p-value	Imputed aOR^i^ (95% CI)	p-value
Any-HPV^f^ + Any-HPV	162	6/34	0.80 (0.33–1.94)	0.621	0.68 (0.27–1.71)	0.409	0.68 (0.27–1.72)	0.417
HR-HPV^g^ + Any-HPV	130	5/35	0.83 (0.25–2.17)	0.912	0.72 (0.26–1.94)	0.510	0.73 (0.27–1.97)	0.533
HPV16 + Any-HPV	36	2/38	1.28 (0.14–5.29)	0.966	1.39 (0.31–6.28)	0.670	1.42 (0.32–6.43)	0.647
NEWBORNS SMALL FOR GESTATIONAL AGE								
	Results from complete case analysis (*N* = 831)						Results from imputed analysis (*N* = 949)	
*N* = 949^a^	HPV positive	SGA^j^ case (HPV pos/neg) *N* = 67	Crude OR (95% CI)	p-value	aOR^k^ (95% CI)	p-value	Imputed aOR^l^ (95% CI)	p-value
Any-HPV^f^ + Any-HPV	171	12/55	0.99 (0.52–1.90)	0.981	1.07 (0.52–2.15)	0.869	0.87 (0.45–1.69)	0.677
HR-HPV^g^ + Any-HPV	139	8/59	0.78 (0.36–1.66)	0.517	0.77 (0.33–1.77)	0.531	0.67 (0.31–1.45)	0.308
HPV16 + Any-HPV	38	1/66	0.35 (0.01–2.13)	0.470	0.37 (0.05–2.78)	0.331	0.28 (0.04–2.08)	0.212

^a^Eight women with missing HDP data, 47 women with missing GDM data and 1 woman with missing SGA data

^b^Hypertensive disorders of pregnancy, including gestational hypertension, superimposed preeclampsia, preeclampsia, eclampsia, Hemolysis, Elevated Liver enzymes, Low Platelets (HELLP)

^c^Adjusted for: Duration of pre-pregnancy relation to child’s father, maternal age, pre-pregnancy Body Mass Index (ppBMI), parity, maternal chronic disease (including chronic hypertension and diabetes mellitus), maternal education

^d^Imputed variables: Maternal education, Duration of pre-pregnancy relation to child’s father, ppBMI

^e^HR-HPV: 16, 18, 31, 33, 35, 39, 45, 51, 52, 56, 58, 59

^f^Any-HPV: 16, 18, 31, 33, 35, 39, 45, 51, 52, 56, 58, 59, 26, 53, 66, 68, 69, 70, 73, 82, 6, 11, 40, 42, 43, 44, 54, 61

^g^GDM: Gestational Diabetes Mellitus

^h^Adjusted for: Maternal Age, ppBMI

^i^Imputed variables: ppBMI

^j^SGA-Newborns small for gestational age

^k^Adjusted for: Maternal chronic disease (including chronic hypertension and diabetes mellitus), nicotine use during pregnancy, alcohol consumption during pregnancy, maternal age. maternal education

^l^Imputed variables: Maternal education, nicotine use during pregnancy, alcohol consumption during pregnancy

Abbreviations: HPV-Human papillomavirus, HR-HPV-High-Risk Human papillomavirus, OR-Odds Ratio, aOR-adjusted Odds Ratio, pos-positive, neg-negative

**Table 4:** Persistent HPV infection from mid-gestation to delivery and adverse pregnancy outcomes

HYPERTENSIVE DISORDERS OF PREGNANCY^a^								
	Results from complete case analysis						Results from imputed analysis	
HPV pos. at mid-gestation	HPV persistent	HDP^b^ case (HPV pos/neg)	Crude OR (95% CI)	p-value	aOR^c^ (95% CI)	p-value	Imputed aOR^d^ (95% CI)	p-value
Any-HPV^e^ *N* = 290	152	11/9	1.12 (0.45–2.79)	0.810	0.95 (0.27–3.32)	0.933	0.99 (0.36–2.73)	0.977
HR-HPV^f^ *N* = 178	93	10/5	1.92 (0.63–5.89)	0.249	3.39 (0.58–19.79)	0.175	1.96 (0.54–7.12)	0.308
HPV16 *N* = 45	29	3/0						
GESTATIONAL DIABETES MELLITUS								
	Results from complete case analysis						Results from imputed analysis	
HPV pos. at mid-gestation	HPV persistent	GDM^g^ case (HPV pos/neg)	Crude OR (95% CI)	p-value	aOR^h^ (95% CI)	p-value	Imputed aOR^i^ (95% CI)	p-value
Any-HPV^e^ *N* = 271	143	3/3	0.91 (0.12–6.88)	1.000	0.81 (0.16–4.27)	0.801	0.81 (0.15–4.26)	0.799
HR-HPV^f^ *N* = 166	88	2/3	1.38 (0.15–16.91)	1.000	1.18 (0.19–7.69)	0.866	1.19 (0.18–7.79)	0.853
HPV16 *N* = 45	29	0			-	-	-	-
NEWBORNS SMALL FOR GESTATIONAL AGE								
	Results from complete case analysis						Results from imputed analysis	
HPV pos. at mid-gestation	HPV persistent	SGA^k^ case (HPV pos/neg)	Crude OR (95% CI)	p-value	aOR^l^ (95% CI)	p-value	Imputed aOR^m^ (95% CI)	p-value
Any-HPV^e^ *N* = 290	152	10/7	1.32 (0.48–3.56)	0.586	1.26 (0.42–3.73)	0.681	1.20 (0.44–3.34)	0.722
HR-HPV^f^ *N* = 178	93	3/5	0.53 (0.12–2.30)	0.400	0.51 (0.10–2.59)	0.417	0.49 (0.11–2.24)	0.356
HPV16 *N* = 45	29	2/0			-	-	-	-

^a^Five women with missing HDP data and 42 women with missing GDM data

^b^Hypertensive disorders of pregnancy, including gestational hypertension, superimposed preeclampsia, preeclampsia, eclampsia, Hemolysis, Elevated Liver enzymes, Low Platelets (HELLP)

^c^Adjusted for: Duration of pre-pregnancy relation to child’s father, maternal age, pre-pregnancy Body Mass Index (ppBMI), parity, maternal chronic disease (including chronic hypertension and diabetes mellitus), maternal education

^d^Imputed variables: Maternal education, Duration of pre-pregnancy relation to child’s father, ppBMI

^e^Any-HPV: 16, 18, 31, 33, 35, 39, 45, 51, 52, 56, 58, 59, 26, 53, 66, 68, 69, 70, 73, 82, 6, 11, 40, 42, 43, 44, 54, 61

^f^HR-HPV: 16, 18, 31, 33, 35, 39, 45, 51, 52, 56, 58, 59

^g^GDM: Gestational Diabetes Mellitus

^h^Adjusted for: Maternal Age, ppBMI

^i^Imputed variables: ppBMI

^j^∞Infinite value

^k^SGA-Newborns small for gestational age

^l^Adjusted for: Maternal chronic disease (including chronic hypertension and diabetes mellitus), nicotine use during pregnancy, alcohol consumption during pregnancy, maternal age. maternal education

^m^Imputed variables: Maternal education, nicotine use during pregnancy, alcohol consumption during pregnancy

Abbreviations: HPV-Human papillomavirus, HR-HPV-High-Risk Human papillomavirus, OR-Odds Ratio, aOR-adjusted Odds Ratio, pos-positive, neg-negative

^n^Fisher’s Exact Test

## Discussion

In this general population cohort of 950 pregnant women, the detection of Any-HPV, HR-HPV, HPV16 or multiple HPV infections at mid-gestation or persistent HPV infections during pregnancy was not associated with increased risk for hypertensive disorders of pregnancy, gestational diabetes mellitus, or newborns small for gestational age, in spite of a relatively high prevalence of HPV infections found during pregnancy.

In our analyses we found no association between HPV infections and HDP, in line with several other studies [4, 8, 27–29]. In contrast, McDonnold et al. who investigated 314 women with presumed HR-HPV infections at the time of entry to prenatal care, matched to 628 women with at least 2 negative consecutive PAP smears, found an association between HPV infections and preeclampsia. However, their study was retrospective, using abnormal PAP smear as a proxy for HR-HPV infections. Further HPV testing was performed on the same sample only if atypical squamous cells of undetermined significance (ASCUS) were found [25]. The true HPV prevalence was not known as women with HPV infection and normal PAP smear were excluded in the exposure group. Our finding of no association between GDM and HPV infections is in line with other studies [25, 27, 28]. Cho et al. surveyed 311 women in Korea six weeks postpartum and tested for 13 h-HPV genotypes. However, as this was done six weeks after delivery, clearance of HPV infections might have occurred yielding fewer HPV positive women postpartum than during pregnancy [28]. Pandey and colleagues however used a prospective design, but in their sample size of 104 women, HPV was detected from condoms used on vaginal ultrasound probes during the first trimester, yielding uncertain results as all women with HPV may not have been identified and this method is yet to be validated [27]. No association was seen between HPV infections and SGA, in line with several other studies [25, 27, 29]. Ford et al. showed an association between HPV and SGA in a study using positive PAP smear 2 years prior to the index pregnancy as a proxy for HPV infection [26]. Similar to the McDonnold study [25], the true HPV prevalence was not known for the current pregnancy. Comparability of our study to others is challenging, as Pandey et al. did not define SGA and McDonnold et al. did not record actual birthweight but rather ultrasound measurements [25, 27]. A systematic review and meta-analysis from 2020 found an association between HPV infections during pregnancy and SGA [24]. However, a true comparison to our present study is difficult as the definition of SGA in the included studies was either missing or varying, ranging from < 10% and < 5% in birthweight percentiles [24].

This current study showed no evidence for increased risk of placental dysfunction disorders including hypertensive disorders of pregnancy, gestational diabetes mellitus or newborns small for gestational age in women with HPV infections compared to women without infections during pregnancy. These findings should be reassuring for pregnant women or women of childbearing age with a positive HPV-screening test result and normal cytology findings. A positive HPV result could reflect an adequate immunological status of the pregnant woman. We lack first trimester HPV status, but as HPV infections take months to be controlled by the immune response, we believe that most HPV infections present in the first trimester are detectable at mid-gestation. It is also possible that most HPV infections do not ascend into the invading trophoblasts early in pregnancy and thereby placentation may not be affected. In this study we have investigated the association of HPV infections and adverse pregnancy outcomes, and not ongoing or previous abnormal cervical cytology due to HPV. This study utilized a prospective design and tested for the most common HPV genotypes at two time points during pregnancy enabling detection of longitudinal HPV infection throughout pregnancy. The effect estimates seem to show no associations, however due to the large uncertainty because of low numbers of HPV infections in women with adverse pregnancy outcomes and low prevalence of adverse pregnancy outcomes, results need to be interpreted carefully. There is a growing body of evidence showing that HPV infections during pregnancy do not affect pregnancy outcomes negatively [8, 27–29] including our present study. Girls who have received the HPV vaccine are now reaching childbearing age, and it will be of great interest to investigate whether the vaccine contributes to further lowering of adverse pregnancy outcomes. In addition to this, more and more countries are implementing HPV testing in the cervical screening programs. With this, women of childbearing age are made aware of their HPV status. This may result in increased social stigma and distress for the pregnant HPV positive women. It is therefore important to inform women that HPV is the most common infection in fertile women and in most cases is a transient infection. The results of this study will further reassure pregnant women and their healthcare workers in charge of their care that a positive HPV result per se will most likely not affect the pregnancy and their unborn fetus pertaining to the adverse pregnancy outcomes investigated in this study.

A strength of this study is the multi-center prospective and longitudinal cohort design. Pregnant women from the general population in Norway and Sweden were invited to participate and biological samples were collected at two time points during pregnancy, allowing a longitudinal HPV infection follow-up. Maternal characteristics were collected through detailed e-questionnaires and medical records. Urine samples were handled according to strict protocols, ensuring high quality samples. First-void urine samples have been shown to be adequate and reliable for detecting genital HPV infections [38, 39]. A weakness of this study is the lack of data concerning HPV status prior to pregnancy and previous treatment for cervical dysplasia, as well as the HPV vaccine status of the women. However, due to their age, most women in the study were likely not vaccinated, as they were not eligible for the HPV vaccine through the childhood vaccine program starting 2009 in Norway and 2010 in Sweden. In addition to this, the first testing timepoint for HPV in this study was in the second trimester, thus HPV status during the first trimester is missing. As the adverse pregnancy outcomes investigated in this study are ongoing processes during the pregnancy, the exact timeline of when the HPV possibly negatively affects the pregnancy is unknown. However, the adverse pregnancy outcomes were recorded at delivery and thereby a causal link between HPV and adverse pregnancy outcomes cannot be made. In our study, we used SGA as a proxy for fetal growth restriction. The distinction between SGA and fetal growth restriction can only be made with ultrasound examinations during pregnancy [40]. We may therefore have included constitutionally small infants amongst those with true placental pathology. Due to the design of this current study, women with miscarriages prior to enrollment at mid-gestation were excluded, preventing us from exploring the possible association between HPV infections and early miscarriage and leading to a potential selection bias when assessing adverse pregnancy outcomes (survival bias). The women enrolled in this study were highly educated and had few comorbidities leading to selection bias. Despite this, we believe the study sample is representative of the general population. For instance, the prevalence of GDM in our study population (4%) was similar to the national prevalence at the time of study period (5%) [41]. We are aware of potential bias due to unmeasured confounding factors. Using the covariates available to us, DAGs were made, and they are presented to show transparency behind the selected covariates. We cannot rule out that there are unmeasured confounding factors that could potentially bias the results.

## Conclusion

In a general population of pregnant women from Norway and Sweden, we found no evidence for an increased risk of adverse pregnancy outcomes linked to placental dysfunction in women with HPV infections during pregnancy, despite the high prevalence of HPV infections observed. These results should be reassuring for women of childbearing age who have been made aware of their HPV status in HPV based screening programs.

## Electronic supplementary material

Below is the link to the electronic supplementary material.

Supplementary Material 1

## Data Availability

The datasets used and/or analysed during the current study are available from the principal investigator in the PreventADALL study on reasonable request. (https://www.oslo-universitetssykehus.no/avdelinger/barne-og-ungdomsklinikken/preventadall/)

## Abbreviations

HPV: Human papillomavirus
HR-HPV: High-Risk Human papillomavirus
HDP: Hypertensive disorders of pregnancy
GDM: Gestational diabetes mellitus
SGA: Small for gestational age
GW: Gestational week
CI: Confidence interval
Aor: Adjusted odds ratio
PreventADALL: Preventing Atopic Dermatitis and ALLergies in children
