# Supplementary material for: Human papillomavirus infections during pregnancy and adverse pregnancy outcomes: a Scandinavian prospective mother-child cohort study
Source: BMC Pregnancy Childbirth. 2024 Nov 19;24:764. doi: 10.1186/s12884-024-06958-2 (PMC11575420; doi:10.1186/s12884-024-06958-2)
Supplement: Supplementary file 1 — Supplementary Material 1 [file 12884_2024_6958_MOESM1_ESM.docx]

**Supplementary Table**

**Supplementary Table 1**: Univariable exact logistic regression models in cells <5.

|  | HPV infections at mid-gestation | | | | |
| --- | --- | --- | --- | --- | --- |
| HYPERTENSIVE DISORDERS OF PREGNANCY | | | | | |
|  | Results from complete case analysis  (N=731) | | | | |
| N= 942^a^ | HPV positive | HDP^b^ case  (HPV pos/neg)  N=83 | | Crude OR  (95% CI) | p-value |
| HPV16 | 59 | 5/78 | | 0.97 (0.29-2.50) | 1.000 |
| GESTATIONAL DIABETES MELLITUS | | | | | |
|  | Results from complete case analysis  (N=883) | | | | |
| N=903^a^ | HPV positive | GDM^c^ case  (HPV pos/neg)  N=40 | | Crude OR  (95% CI) | p-value |
| HPV16 | 57 | 2/38 | | 0.77 (0.09-3.13) | 1.000 |
| NEWBORNS SMALL FOR GESTATIONAL AGE | | | | | |
|  | Results from complete case analysis  (N=831) | | | | |
| N=949^a^ | HPV positive | SGA^d^ case  (HPV pos/neg)  N=67 | | Crude OR  (95% CI) | p-value |
| HPV16 | 59 | 2/65 | | 0.45 (0.05-1.75) | 0.390 |
| Multiple HPV infections at mid-gestation | | | | | |
| HYPERTENSIVE DISORDERS OF PREGNANCY | | | | | |
|  | Results from complete case analysis  (N=731) | | | | |
| N=942^a^ | HPV  positive | HDP^b^ case  (HPV pos/neg)  N=83 | | Crude  OR  (95% CI) | p-value |
| HPV16 + Any-HPV^e^ | 38 | 4/79 | | 1.23  (0.31-3.58) | 0.870 |
| GESTATIONAL DIABETES MELLITUS | | | | | |
|  | Results from complete case analysis  (N=731) | | | | |
| N=903^a^ | HPV  positive | GDM^c^ case  (HPV pos/neg)  N=40 | | Crude  OR  (95% CI) | p-value |
| HPV16 + Any-HPV^e^ | 36 | 2/38 | | 1.28  (0.14-5.34) | 0.963 |
| NEWBORNS SMALL FOR GESTATIONAL AGE | | | | | |
|  | Results from complete case analysis  (N=731) | | | | |
| N=949^a^ | HPV  positive | SGA^d^ case  (HPV pos/neg)  N=67 | | Crude  OR  (95% CI) | p-value |
| HPV16 + Any-HPV^e^ | 38 | 1/66 | | 0.35  (0.01-2.12) | 0.469 |
| Persisting infections from mid-gestation to delivery | | | | | |
| HYPERTENSIVE DISORDERS OF PREGNANCY | | | | | |
|  | Results from complete case analysis | | | | |
| HPV pos. at mid-gestation | HPV persistent | | HDP^b^ case  (HPV pos/neg) | Crude  OR  (95% CI) | p-value |
| HPV16  N=45 | 29 | | 3/0 |  |  |
| GESTATIONAL DIABETES MELLITUS | | | | | |
|  | Results from complete case analysis | | | | |
| HPV pos. at mid-gestation | HPV persistent | | GDM^c^ case  (HPV pos/neg) | Crude  OR  (95% CI) | p-value |
| Any-HPV^e^  N=290 | 152 | | 3/3 | 0.89  (0.12-6.79) | 1.000 |
| HR-HPV^f^  N=178 | 93 | | 2/3 | 1.34  (0.15-16.43) | 1.000 |
| HPV16  N=45 | 29 | | 0 | ^-^ | ^-^ |
| NEWBORNS SMALL FOR GESTATIONAL AGE | | | | | |
|  | Results from complete case analysis | | | | |
| HPV pos. at mid-gestation | HPV persistent | | SGA^d^ case  (HPV pos/neg) | Crude  OR  (95% CI) | p-value |
| HPV16  N=45 | 29 | | 2/0 | - | ^-^ |

^a^Five women with missing HDP data and 42 women with missing GDM data.

^b^Hypertensive disorders of pregnancy, including gestational hypertension, superimposed preeclampsia, preeclampsia, eclampsia, Hemolysis, Elevated Liver enzymes, Low Platelets (HELLP).

^c^GDM: Gestational Diabetes Mellitus.

^d^SGA-Newborns small for gestational age.

^e^Any-HPV: 16, 18, 31, 33, 35, 39, 45, 51, 52, 56, 58, 59, 26, 53, 66, 68, 69, 70, 73, 82, 6, 11, 40, 42, 43, 44, 54, 61

^f^HR-HPV: 16, 18, 31, 33, 35, 39, 45, 51, 52, 56, 58, 59

Abbreviations: HPV-Human papillomavirus, HR-HPV-High-Risk Human papillomavirus, OR-Odds Ratio, pos-positive, neg-negative.

**Supplementary Figure**

**
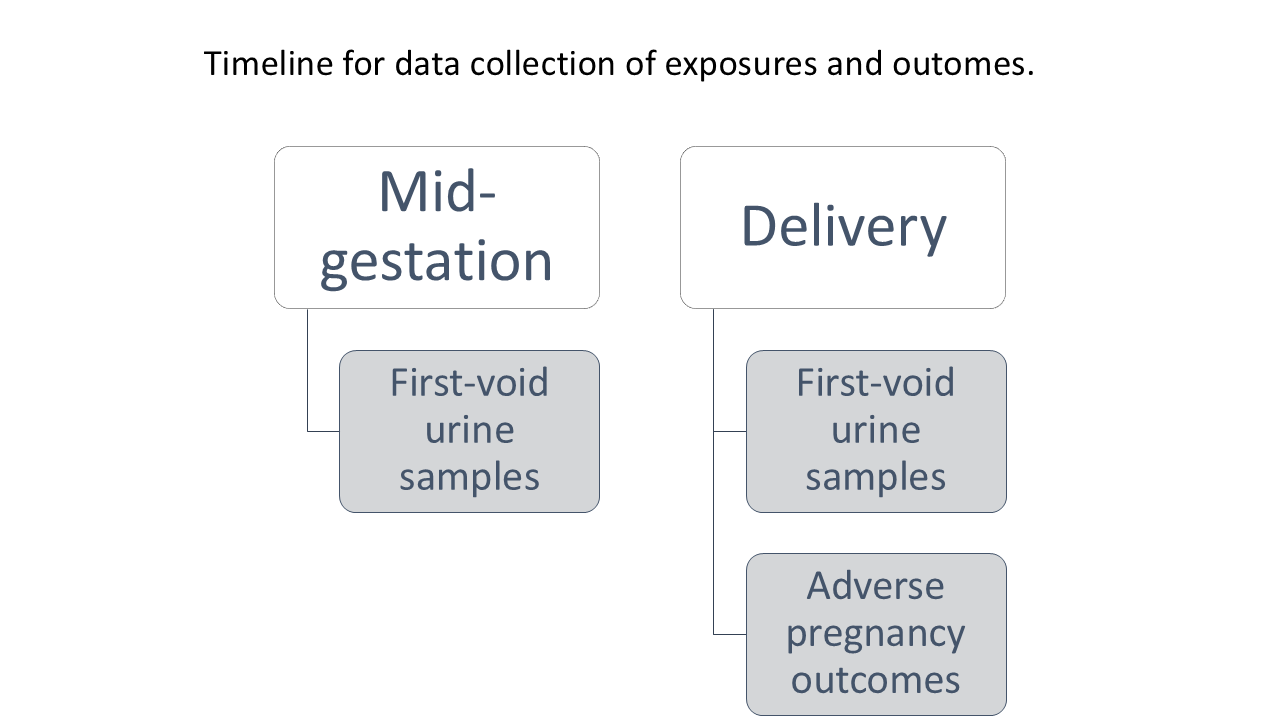
**
